# Coexistence of Monoclonal Gammopathy and Hemolysis in Chronic Lymphocytic Leukemia: Unveiling a Rare Case

**DOI:** 10.7759/cureus.69766

**Published:** 2024-09-19

**Authors:** Poornima Pandey, Ankita Gyanchandani, Navneet Singh Saluja, Shreya Giri Goswami, Lokesh Singh Chauhan

**Affiliations:** 1 Department of Pathology, Datta Meghe Institute of Higher Education and Research, Wardha, IND; 2 Department of Orthopedics, Datta Meghe Institute of Higher Education and Research, Wardha, IND

**Keywords:** b-cells, chronic lymphocytic leukemia (cll), hemolysis, monoclonal gammopathy, serum protein electrophoresis (spep)

## Abstract

In B-cell malignancies, there is a periodic presence of monoclonal gammopathies. In a notable multitude of cases with chronic lymphocytic leukemia (CLL), a cluster of antigen-inciting B-cells sometimes shows the presence of monoclonal gammopathy and autoimmune hemolysis simultaneously. The detection of monoclonal proteins or light chains in urine and/or serum is significantly increased in cases of CLL and can be identified using highly sensitive laboratory methods, such as serum protein electrophoresis. Hemolysis in these patients can be detected by the direct Coombs test (DCT).

Several scientific research data indicate that the findings of hemolysis and the presence of monoclonal proteins have an adverse impact on the survival of patients. Nevertheless, there is no perspicuous proof to indicate the prognostic importance of hemolysis and monoclonal gammopathy in patients with CLL, even though monoclonal proteins and hemolysis in CLL generally occur at prevalences of 60%-80% and 5%-10%, respectively, very few cases have been reported in the literature. Owing to the peculiarity, we report a case of CLL diagnosed in 2023. The 66-year-old woman had developed the progressive disease along with the existence of monoclonal gammopathy and hemolysis. Although the manifestation of both findings could be due to the use of highly sensitive methods, it may also be attributable to an autoimmune process or progression from similar or distinct B-cell clones.

## Introduction

The antigen-stimulated B-cells by escaping programmed cell death give rise to a disorder of chronic lymphocytic leukemia (CLL) [1]. The B-cell malignancies are known to show monoclonal gammopathy at frequent intervals. As compared to the conventional techniques the appearance of monoclonal paraproteins and hemolysis in conjunction with CLL is more commonly seen with the usage of highly sensitive techniques. The autoimmune cause can be considered, as CLL B-cells can act just like the dendritic cells, propelling self-exciting T-helper cells and potentially prompting T-cell subset disparity, which may advocate auto-reactive B lymphocytes that generate anti-RBC autoantibodies [2]. There is a noteworthy female preponderance, with a ratio of 2:1 compared to the male population. The literary sources that describe the diagnosis of CLL in conjunction with monoclonal gammopathy and hemolysis are rare [3].

Therefore to analyze the remarkable morphological medical diagnosis, we present a rare case of a 66-year-old woman detected with a lymphoid disorder, inculcated paraproteinemia in association with advancing hemolysis in the form of erythrocytopenia, pleural effusion, and swollen lymph node.

## Case presentation

**Figure 1:**
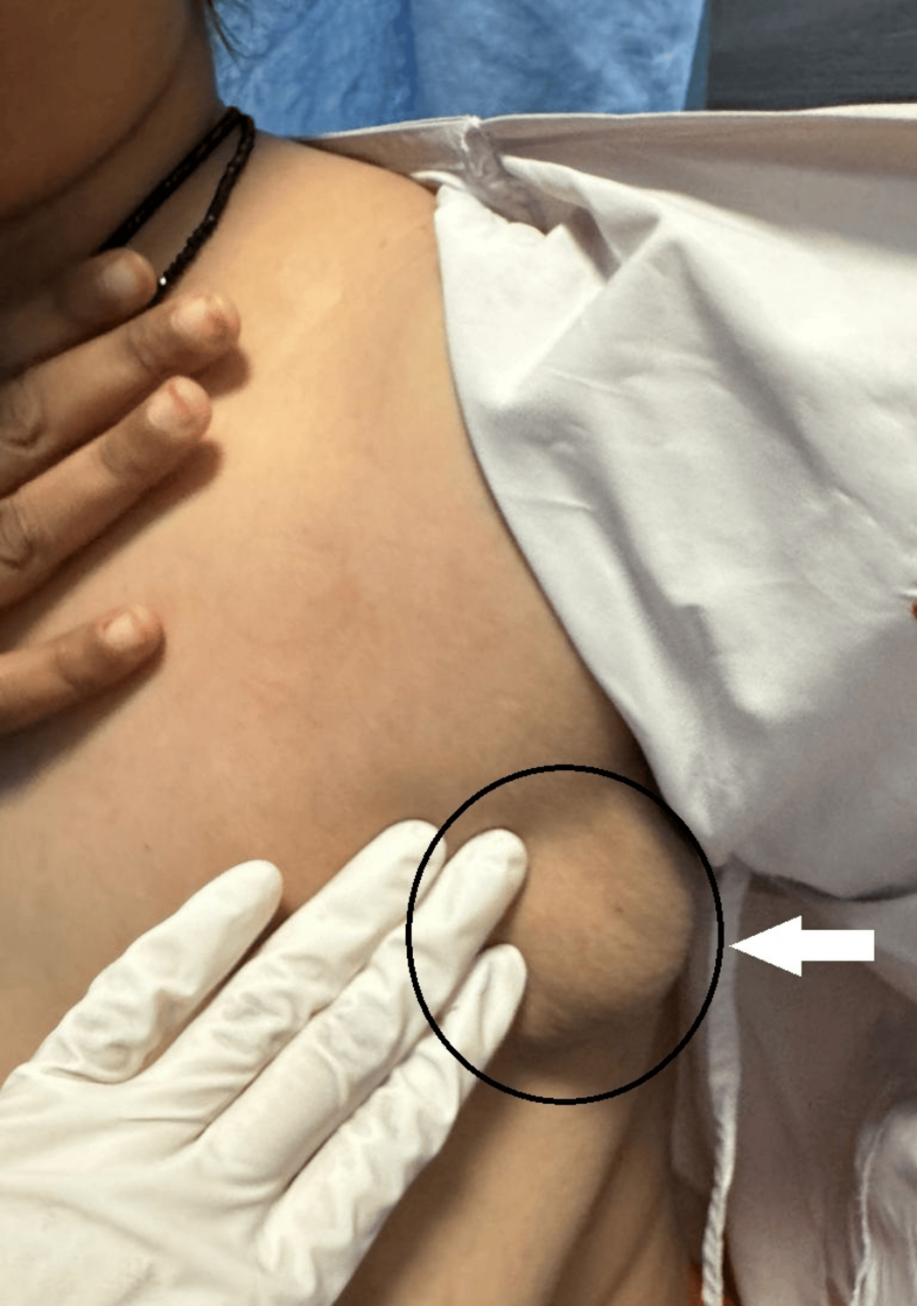
Clinical photograph showing left axillary lymphadenopathy with the circle and arrow pointing to a prominent bulge

A 66-year-old female patient presented with the chief complaints of cough, breathlessness, generalized weakness, and evening rise of temperature for 15 days. She presented with left axillary swelling approximately the size of a lemon, which appeared two months back. The swelling was initially subtle and progressive in size. The overlying skin seemed to be unremarkable along with a prominent bulge (Figure 1).

The clinical examination displayed a palpable firm and non-tender left axillary lymphadenopathy. Her routine investigations owing to the diagnostic relevance were carried out (Table 1).

**Table 1:** Laboratory investigations of the patient TLC: total leucocyte count; LDH: lactate dehydrogenase; SGOT: serum glutamic-oxaloacetic transaminase; SGPT: serum glutamic pyruvic transaminase; CRP: C-reactive protein

Parameters	Patient’s test results	Unit of measurement	Normal reference values
Hemoglobin	7.3	g/dL	12.0-15.0 g/dL
TLC	2,88,000	cells/cu.mm	4,500-11,000 cells/cu.mm
Platelet count	2,27,000	platelets/microliter of blood	1,50,000-4,50,000 platelets/microliter of blood
LDH	172	U/L	140-280 U/L
Serum uric acid	6.8	mg/dL	3.5-7.2 mg/dL
Serum creatinine	1.3	mg/dL	0.6-1.1 mg/dL
Total bilirubin	1.4	mg/dL	0.1-1.2 mg/dL
SGOT	83	U/L	8-45 U/L
SGPT	67	U/L	7-56 U/L
CRP	1.75	mg/dL	0.8-1.0 mg/dL
Reticulocyte count	4.8	%	0.5%-2.5%

**Figure 2:**
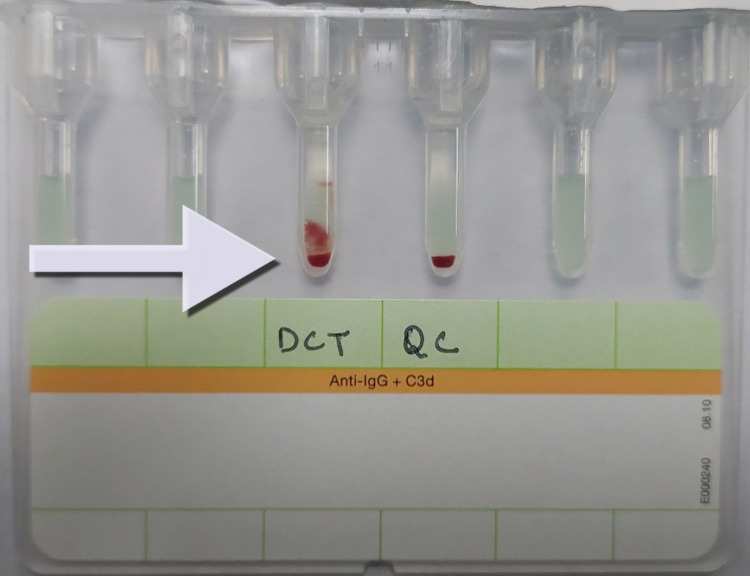
Gel card showing the positive result of DCT and arrow showcasing Grade 2+ reaction DCT: direct Coombs test; QC: quality control

The complete blood picture revealed that the patient had plunged within the range of anemia. The total leucocyte count (TLC) was elevated along with lymphocytosis. The lactate dehydrogenase (LDH) and serum uric acid levels were within the standard range. The values of total bilirubin and the reticulocyte count were indicative of ongoing hemolysis. The direct Coombs test (DCT) of the patient was positive showing a Grade 2+ reaction (Figure 2).

**Figure 3:**
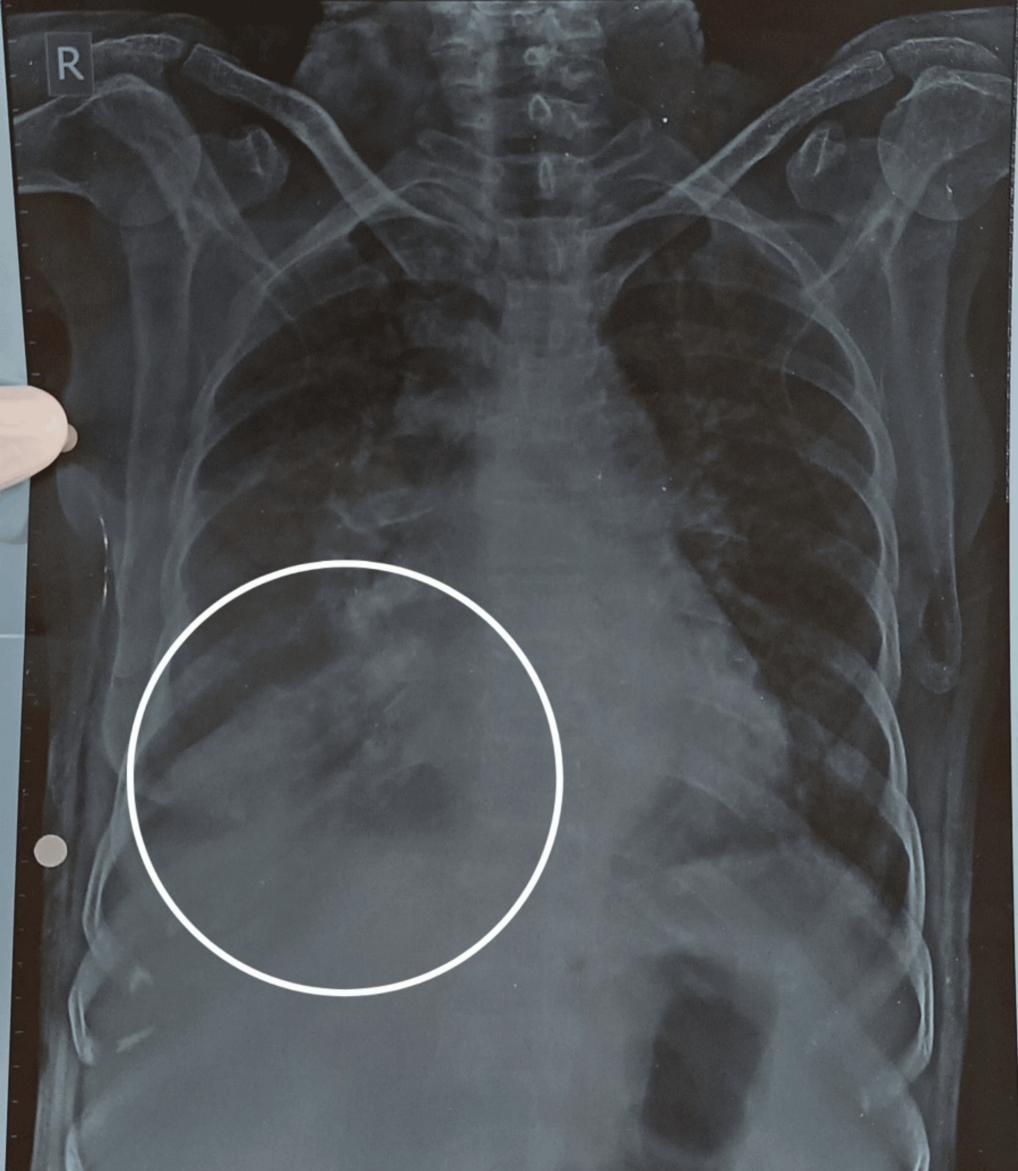
Right-sided chest radiograph PA view showing mild pleural effusion. The circular area depicts increased homogenous density near the costophrenic angle PA: posterior anterior

The probable cause of it may be the suspected self-antigens deflected against red blood cells with or without complement system stimulation. Since the patient had a persistent cough, the posterior-anterior (PA) chest radiograph was taken, which depicted mild pleural effusion on the right side (Figure 3).

**Figure 4:**
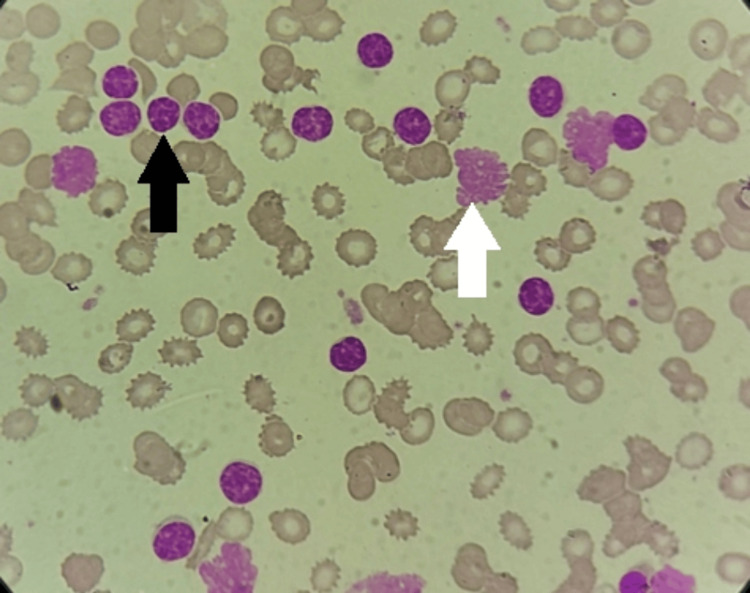
Peripheral smear showing "soccer ball" morphology of lymphocytes (black arrow) and smudge cells (white arrow), seen in CLL CLL: chronic lymphocytic leukemia

The hemato-morphological features observed on the peripheral smear of the patient illustrated lymphocytosis. There were numerous small mature lymphocytes and smudge cells (Figure 4).

**Figure 5:**
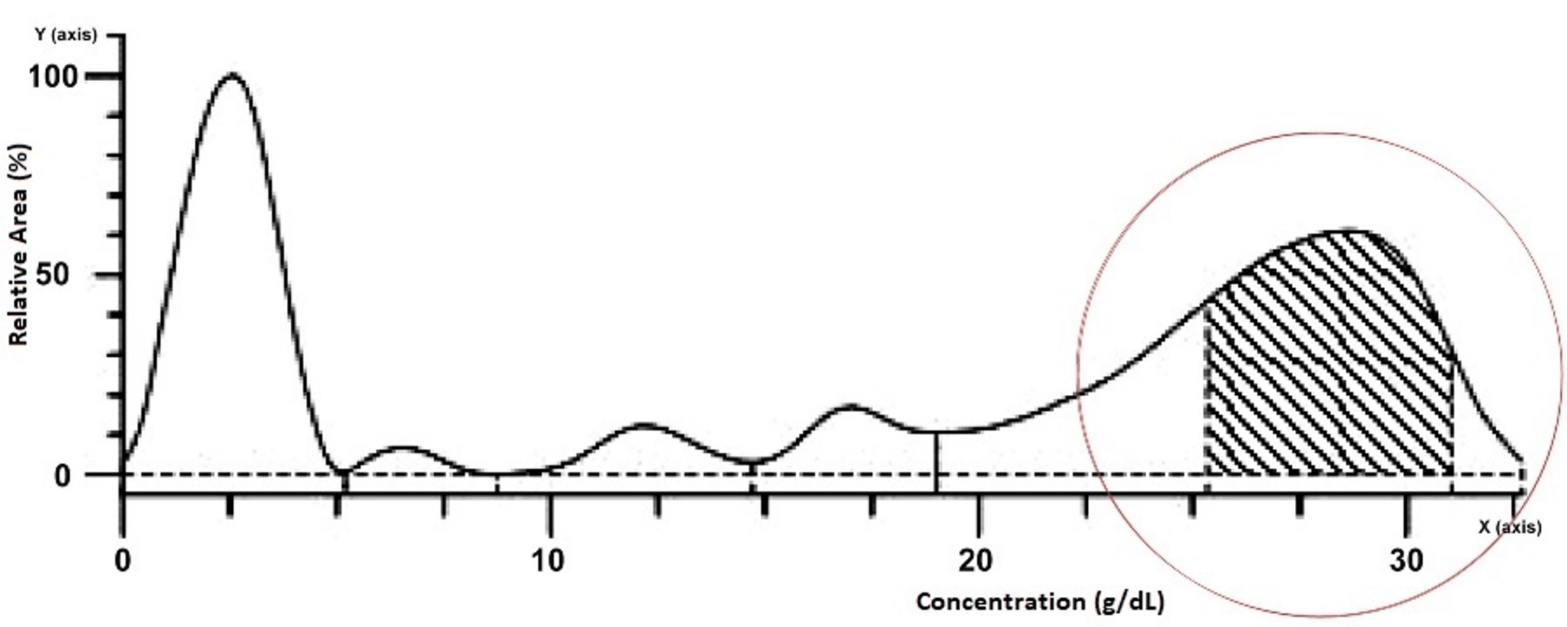
Graphical representation of serum protein electrophoresis showing "M"-band in gamma globulin region (shaded area), X-axis depicting concentration in g/dL and Y-axis depicting relative area in % M: monoclonal

The lymphocytes had a "soccer ball" morphology with a mature dense nucleus having a "cracked appearance" indicative of the diagnosis of CLL. The serum protein electrophoresis test revealed a uniform peak in the prime area of the gamma-globulin region depicting the "M" band ("M"-monoclonal) (Figure 5).

The test results were also tabulated illustrating a concentration of 3.29 H and 36.66% relative area of gamma-globulin protein (Table 2).

**Table 2:** Test results of serum protein electrophoresis highlighting relative area and salient concentration of gamma (M-band) M: monoclonal

Index	Band	Relative area	Concentration (g/dL)	Range (g/dL)
1.	Albumin	32.16%	2.88 L	3.50-5.00
2.	Alpha 1	1.47%	0.13 L	0.10-0.40
3.	Alpha 2	4.21%	0.38 L	0.50-1.10
4.	Beta	5.85%	0.53 L	0.60-1.30
5.	Gamma-globulin	56.30%	5.05 H	0.60-1.60
Results	Gamma M	36.66%	3.29 H	
Total protein			8.97 g/dL	
Albumin/Globulin (A/G) ratio			0.47	

The other test values including albumin fraction, α-1 globulin, α-2 globulin, β globulin, and A/G ratio were within reference ranges except for the γ-globulin value of 5.05 g/dL (Table 3).

**Table 3:** Tabulated serum protein values comparable to gamma M-spike M: monoclonal

Protein electrophoresis	Result	Units	Reference range
Albumin fraction	2.88	g/dL	3.50-5.00
Alpha 1-globulin	0.13	g/dL	0.10-0.40
Alpha 2-globulin	0.38	g/dL	0.50-0.10
Beta globulin	0.53	g/dL	0.60-1.30
Gamma-globulin	5.05	g/dL	0.60-1.60
Protein-total (method: Biuret)	8.97	g/dL	6.00-8.00
Albumin/globulin ratio	0.47	g/dL	1.0-2.1
Gamma M-spike	3.29		

The urine for Bence-Jones (BJ) proteins was negative. The patient was advised to acquire further follow-up with two weeks of ibrutinib treatment along with cytogenetic studies and immunofixation electrophoresis facilitating the prognostication.

## Discussion

CLL is related to enterprising γ-globulin deficiency, which can entail one or another IgG subcategories [3]. The research by Mozas et al. and Al-Riyami et al. have inferred the occurrence of monoclonal immunoglobulin in subjects with CLL arrays from 60% to 80% [4,5]. IgG (κ) is the most prevailing abnormality detected in CLL, followed by IgG (λ) and IgM (λ) in order of prevalence [6]. Free (lambda and kappa) light chains are also one of the recurrently detected abnormalities in CLL. They can occur in 20% to 30% of patients with CLL [7]. The main pathogenesis of the presence of M proteins is not well known, it can be because of immunological aspects or due to deviation from similar or dissimilar clones of B-cells.

The studies of Maurer et al. and Pratt et al. have revealed that free light chain malformations are linked with feeble outcomes in CLL [8,9]. The accurate role of monoclonal or polyclonal proteins in CLL is not comprehensible, although some of the research done by Laurenti et al. has shown that the existence of monoclonal or polyclonal proteins is an impoverished predictive factor [10]. It is challenging to withdraw interpretations from formerly outlined cases with respect to their role in CLL, as there are few reported cases. Additionally, there are no case-controlled literary sources completed regarding the matter. In the condition of CLL, the identification of hemolysis can be tough since blood markers such as hemoglobin, hemolytic markers, and DCT that are pertinent for hemolysis may be altered by CLL development or concurrent treatment [11,12]. From a prognosticative point of view, hemolysis does not remarkably influence the diagnosis [13].

The principal determinant in our current study, monoclonal gammopathy is present in up to 20% of cases of CLL and correlates with a poor prognosis [6]. The additionally mentioned modality of hemolysis is a common complication in CLL, occurring in 10% to 25% of patients during the course of their disease. The DCT may be positive at some time during the disease course in up to 35% of cases, but overt hemolysis occurs less frequently. Although hemolysis may occur in asymptomatic untreated CLL, it is more common in patients with advanced-stage disease [14]. The present case study observed the simultaneous synchronal presence of hemato-morphological features of CLL, monoclonal gammopathy, and hemolysis. The diagnosis of CLL was entertained by virtue of peculiar monomorphy of the lymphoid cell population. The monoclonal gammopathy was overruled using serum protein electrophoresis and the evidence of hemolysis was aided by biochemical parameters as well as DCT. Varied outcomes of the patients with monoclonal gammopathy and hemolysis in various studies and literature make this case peculiar for its inclusion in CLL prognostic stratification.

## Conclusions

Serum paraprotein can be detected in a subset of patients with CLL by serum protein electrophoresis and immunofixation electrophoresis. The WHO classification recognizes that a “small M component” can be found in some patients with CLL, but no mention is made of the frequency of this occurrence or the range of serum paraprotein levels in patients with CLL. Along with these, the DCT status serves as a surrogate marker at the time of initiation of therapy for CLL. Also, it is a new prognostic indicator for both disease-free survival and overall survival. Despite much ongoing research on this distinctive subject matter more updated literary sources and substantiation are essential for a better apprehension of the pathogenesis and usage of the information in the clinico-pathological field for better prognosis. This might aid us in therapeutic resolution in the near future.
